# Evidence for the Existence of a CXCL17 Receptor Distinct from GPR35

**DOI:** 10.4049/jimmunol.1700884

**Published:** 2018-06-06

**Authors:** Nurul A. S. Binti Mohd Amir, Amanda E. Mackenzie, Laura Jenkins, Karim Boustani, Marston C. Hillier, Tomoko Tsuchiya, Graeme Milligan, James E. Pease

**Affiliations:** * Inflammation, Repair and Development Section, National Heart and Lung Institute, Faculty of Medicine, Imperial College London, London SW7 2AZ, United Kingdom;; † Medical Research Council and Asthma UK Centre in Allergic Mechanisms of Asthma, London, United Kingdom;; ‡ Centre for Translational Pharmacology, Institute of Molecular, Cell, and Systems Biology, College of Medical, Veterinary and Life Sciences, University of Glasgow, Glasgow G12 8QQ, United Kingdom; and; § Research Institute, National Center for Global Health and Medicine, Shinjuku-ku, Tokyo 162-8655, Japan

## Abstract

The chemokine CXCL17 is associated with the innate response in mucosal tissues but is poorly characterized. Similarly, the G protein–coupled receptor GPR35, expressed by monocytes and mast cells, has been implicated in the immune response, although its precise role is ill-defined. A recent manuscript reported that GPR35 was able to signal in response to CXCL17, which we set out to confirm in this study. GPR35 was readily expressed using transfection systems but failed to signal in response to CXCL17 in assays of β-arrestin recruitment, inositol phosphate production, calcium flux, and receptor endocytosis. Similarly, in chemotaxis assays, GPR35 did not confirm sensitivity to a range of CXCL17 concentrations above that observed in the parental cell line. We subsequently employed a real time chemotaxis assay (TAXIScan) to investigate the migratory responses of human monocytes and the monocytic cell line THP-1 to a gradient of CXCL17. Freshly isolated human monocytes displayed no obvious migration to CXCL17. Resting THP-1 cells showed a trend toward directional migration along a CXCL17 gradient, which was significantly enhanced by overnight incubation with PGE_2_. However, pretreatment of PGE_2_-treated THP-1 cells with the well-characterized GPR35 antagonist ML145 did not significantly impair their migratory responses to CXCL17 gradient. CXCL17 was susceptible to cleavage with chymase, although this had little effect its ability to recruit THP-1 cells. We therefore conclude that GPR35 is unlikely to be a bona fide receptor for CXCL17 and that THP-1 cells express an as yet unidentified receptor for CXCL17.

## Introduction

Intensive efforts by the chemokine research community over the last two decades have identified a family of around 45 such proteins in the human, noted for their ability to induce the directional migration (i.e., chemotaxis) of leukocytes (1). Considerable progress has been made regarding our understanding of this family and how the signals they induce via specific G protein–coupled receptors (GPCRs) shape the immune responses of the host (2). In the case of the chemokine receptors CCR5 and CXCR4, this knowledge has been successfully translated into medicines with clinical efficacy in the treatment of HIV infection, the treatment of WHIM (warts, hypogammaglobulinemia, immunodeficiency, and myelokathexis) syndrome, and the mobilization of stem cells (3–5). Despite this progress, within the chemokine family there still remains a small number of orphan chemokines for which no specific GPCR partners have been identified. These include the CXC chemokines CXCL14 (6, 7) and CXCL17 (8).

CXCL17 was first described in the literature as a monocyte-recruiting chemokine (8), and its overexpression has been shown to promote the growth of a variety of tumors in vivo (9, 10). In humans, CXCL17 appears to have roles in both homeostatic and inflammatory settings. Its expression is restricted to mucosal sites, including the small intestine, trachea, and lung, where it is associated with a broad spectrum of antimicrobial function, albeit when at micromolar concentrations of chemokine (11). Notably, CXCL17 was undetectable in the bronchioalveolar lavage of healthy subjects but expressed at significant levels in the bronchioalveolar lavage of patients suffering from idiopathic pulmonary fibrosis (IPF) (11). This prompted the authors of that study to speculate that CXCL17 plays a role in microbial killing within the IPF lung (often associated with infection in advanced stages of the disease) or is involved with the associated remodeling via the recruitment of myeloid cells. Consistent with this latter hypothesis, the same group went on to generate a CXCL17-deficient mouse model that was notable for the reduced levels of macrophages observed in the lung under homeostatic conditions (12).

GPR35 was originally identified in the laboratory of O’Dowd (13) as an open reading frame predicted to encode a GPCR. Subsequent demonstration that it is expressed by various cells of the immune system has led to the suggestion that it may have potential as a therapeutic target in inflammatory disease (14, 15). In human, two distinct GPR35 isoforms known as GPR35a and GPR35b are expressed, with GPR35b differing from GPR35a by the presence of an additional 31 aa at the N terminus (16), analogous to the two N-terminally spliced isoforms of the chemokine receptor CXCR3 (17). Endogenous ligands identified as activating GPR35 include the tryptophan metabolite kynurenic acid (18) and various lysophosphatidic acids (19), although the millimolar concentrations of the former ligand needed to induce signaling at human GPR35 has led to questions about the physiological relevance of the original finding (20). Among synthetic compounds, the asthma medications cromolyn disodium (21) and lodoxamide (22), which serve to stabilize mast cells, have also been shown to be agonists of GPR35, implicating GPR35 in the allergic response.

Recently, Maravillas-Montero et al. (23) described CXCL17 as a GPR35 ligand with nanomolar activity in both chemotaxis and intracellular calcium flux assays. In this article, we describe an investigation into the potency and specificity of CXCL17 as a GPR35 ligand using a battery of in vitro assays in both GPR35 transfected cell lines, human monocytes, and the human myeloid cell line THP-1.

## Materials and Methods

### Materials

Materials were purchased from Thermo Fisher Scientific (Paisley, U.K.) unless otherwise described. Recombinant human CXCL17 generated in *Escherichia coli* was purchased from R&D Systems (Abingdon, U.K.) and was comprised of the 96 aa Leu^24^–Leu^119^. A plasmid containing an insert encoding a 3xHA-tagged GPR35a cDNA (catalog no. GPR035TN00) was purchased from the cDNA Resource Center (Bloomsburg University, Bloomsburg, PA) and is an N-terminal 3xHA-tagged variant of the GPR35 plasmid that was derived by PCR from the clone used by Maravillas-Montero et al. (23). The insert was resequenced by Eurofins MWG (Ebersberg, Germany) and found to be authentic. Other cDNAs encoding human GPR35a and GPR35b as well as the mouse and rat orthologs of GPR35 have been detailed previously (20). The GPR35 antagonist ML145 was purchased from Bio-Techne (Oxford, U.K.) and was originally identified as a GPR35 inhibitor by a group from the Sanford-Burnham Center for Chemical Genomics using an arrestin recruitment assay (bioassay identifier: 2079) (24). The murine anti-hemagglutinin (HA) mAb (HA.11) was purchased from Cambridge Bioscience (Cambridge, U.K.). CCL2, CCL15, and CCL23 were purchased from PeproTech EC (London, U.K.)

### Cell culture

The mouse pre–B cell line L1.2 and THP-1 cells were cultured in RPMI 1640 (Sigma-Aldrich, Dorset, U.K.) containing 10% FBS, penicillin/streptomycin, MEM nonessential amino acids, sodium pyruvate, and 2-ME and incubated at 37°C, 5% CO_2_. Cells were maintained in this medium at a concentration of 0.5 × 10^6^ cells/ml. Human embryonic kidney (HEK) 293 T cells were maintained in DMEM supplemented with 0.292 g/l l-glutamine, 10% (v/v) FBS, and 1% penicillin/streptomycin mixture. Peripheral blood was taken from healthy normal subjects with informed consent according to a protocol approved by a local ethics committee. Monocytes were isolated by negative selection using the RosetteSep kit (StemCell Technologies, Grenoble, France).

### Cell transfections

This protocol was essentially that as previously described (25). Briefly, 1.0–1.5 × 10^7^ cells/ml were used for each transfection in a volume of 800 μl of RPMI 1640. This was transferred to a 0.4-cm gap electroporation cuvette, after which 50 μl of a 10 mg/ml solution of baker’s yeast tRNA (Sigma-Aldrich) was added to the cuvette. One microgram of the GPR35 plasmid per 1 × 10^6^ cells was added to the cuvette, and the cells were subjected to electroporation at 330 V, 950 μF. After allowing the cells to recover for 20 min at room temperature, cells were transferred to a flask containing complete medium and incubated for 18 to 24 h before receptor expression was examined by flow cytometry prior to experimentation. For transient transfections, sodium butyrate (Sigma-Aldrich) was added to a final concentration of 10 mM to enhance receptor expression. To generate the L1.2 clone 23 stably expressing GPR35, cells were transfected as before and selected after 48 h of culture by the addition of 1 mg/ml G418. Surviving cells were expanded and cloned by limiting dilution. Clone 23 was identified by flow cytometry as expressing suitable amounts of GPR35 on its surface. Transient transfections using HEK293 T cells were performed using polyethylenimine, with experiments carried out 48 h after transfection.

### Flow cytometry

Briefly, 0.5 × 10^6^ transfected cells were used for each staining procedure. Cell pellets were resuspended in 100 μl of FACS buffer (PBS, 0.25% BSA, 0.05% NaN_3_) containing primary Ab at a concentration of 10 μg/ml and incubated for 5 min on ice. The cells were then washed with 500 μl FACS buffer and pelleted by centrifugation (1500 × *g*) for 30 s. The supernatant was discarded, and the cell pellet was resuspended in 100 μl FACS buffer containing FITC-conjugated secondary Ab and incubated at 4°C for 5 min. The cells were then washed with 500 μl FACS buffer and centrifuged briefly as before for 30 s. The supernatant was discarded, and the cell pellets were resuspended in 500 μl staining buffer containing TO-PRO3 at a dilution of 1:10,000. The samples were read on a FACSCalibur flow cytometer (Becton Dickinson, Oxford, U.K.).

### Modified Boyden chamber chemotaxis assays

Assays were performed essentially as described by Vaidehi et al. (25) using 96-well disposable chemotaxis plates (Neuro Probe, Gaitherburg, MD). Cells were allowed to migrate in response to chemokines for 5 h at 37°C, 5% CO_2_, after which CellTiter-Glo (Promega, Southampton, U.K.) was used to measure the responses via luminescence, and responses were read from a plate reader as before (26). Data from these assays are shown as chemotactic indices following division by the basal migratory responses to buffer alone. In some experiments, prior to use in the assay, cells were preincubated for 15 min at room temperature in buffer containing DMSO (vehicle) or a 1 μM final concentration of the GPR35 antagonist ML145.

### Endocytosis assay

L1.2 cells expressing 3xHA-GPR35 were resuspended in RPMI containing 0.1% BSA at 2 × 10^6^ cells/ml. To 25-μl volumes of cells was added 25 μl of the same buffer containing either 20 μM lodoxamide, 10 μM lodoxamide, 2 μM lodoxamide, or 2 μM CXCL17. Controls contained the vehicle DMSO. For each data point, the cells were incubated for 15 min at 4°C (to measure basal GPR35 expression in the absence of endocytosis) and 37°C (to measure agonist-driven endocytosis of the receptor). GPR35 remaining on the cell surface after treatment was quantified by flow cytometry using the anti-HA Ab as described above.

### Real-time chemotaxis assays (TAXIScan)

TAXIScan apparatus for the real time visualization of leukocyte migration was assembled and used according to a published protocol (27). Monocytes or THP-1 cells were allowed to migrate for 1 h at 37°C along gradients generated by the addition of 1 μl of either 1 μM CXCL17, 10 μM CXCL17, or 1 μM CCL2. Cells were also allowed to migrate in the absence of a stimulus to obtain basal chemotaxis measurements. In some experiments, prior to use, cells were preincubated for 15 min at room temperature in buffer containing DMSO (vehicle) or 1 μM final concentration of the GPR35 antagonist ML145. Sequential image data were captured every minute as individual JPEGs, which were subsequently processed with ImageJ (National Institutes of Health) equipped with the manual tracking (Fabrice Cordelieres; Institut Curie, Orsay, France) and chemotaxis tool plugins (ibidi, Martinsried, Germany).

Individual experiments consisted of triplicate or quadruplicate for each stimulus, and data illustrated are collated from an equal number of experiments as highlighted in the appropriate figure legend. The total numbers of cells tracked under each condition are shown in the top right hand corner of each plot. For each individual cell, the forward migration index parallel to the gradient (FMI^‖^) was calculated using the chemotaxis tool plugin. FMI^‖^ is defined as the distance traveled by the cell in the *y*-axis (i.e., along the chemokine gradient) divided by the accumulated distance traveled and is a reliable measure of migration along a chemoattractant gradient (28).

### GPR35–β-arrestin-2 interaction assays

The bioluminescence resonance energy transfer–based β-arrestin-2 recruitment assays were performed using HEK293 T cells transfected transiently to coexpress forms of human, mouse, or rat GPR35 along with β-arrestin-2, as described previously (20).

### Inositol phosphate assays

Chimeric G protein α subunits were as described in Jenkins et al. (20), and, following expression in HEK293 T cells along with the described forms of GPR35, inositol phosphate measurements were performed as previously described (20).

### Intracellular calcium flux assays

These were performed as previously described (29) using cells that were loaded with the dye FURA-2 AM. Real-time data were recovered using a fluorimeter (LS-50B; PerkinElmer, Beaconsfield, U.K.). Data are expressed as the relative ratio of fluorescence emitted at 510 nm after sequential stimulation at 340 and 380 nm.

### Chemokine digestion and SDS-PAGE analysis

Twenty micrograms of CXCL17 was resuspended in a buffer composed of 100 mM Tris-HCl (pH 7.8) and 10 mM CaCl_2_. The aliquot was divided into two, with one aliquot receiving the addition of 0.1–0.4 μg of recombinant human chymase (Sigma-Aldrich, Poole, U.K.). Both tubes were incubated for 18 h at 37°C before being stored at −20°C until further use. SDS-PAGE was carried out using precast NuPAGE 4–12% Bis-Tris Protein Gels. Samples were mixed with 2× Tris-Glycine SDS Sample Buffer and heated to 95°C for 10 min prior to loading alongside SeeBlue Plus2 prestained protein standards. Gels were run at 150 V for 50 min and then fixed in 25% isopropanol and 10% acetic acid (HOAc) for 30 min with agitation. Fixed gels were stained with 0.25% Coomassie Brilliant Blue G-250 (Bio-Rad Laboratories, Watford, U.K.) in 25% isopropanol and 10% HOAc for 2 h, after which they were destained with 10% HOAc overnight. Images were acquired with myECL Imager (Thermo Fisher Scientific).

### Statistical analysis

Data analysis was carried out using GraphPad Prism version 6.0 (GraphPAD Software, San Diego, CA). Experiments were assessed, and appropriate statistical tests were noted in the figure legends. In all tests, *p* < 0.05 was considered as statistically significant.

## Results

**FIGURE 1.**
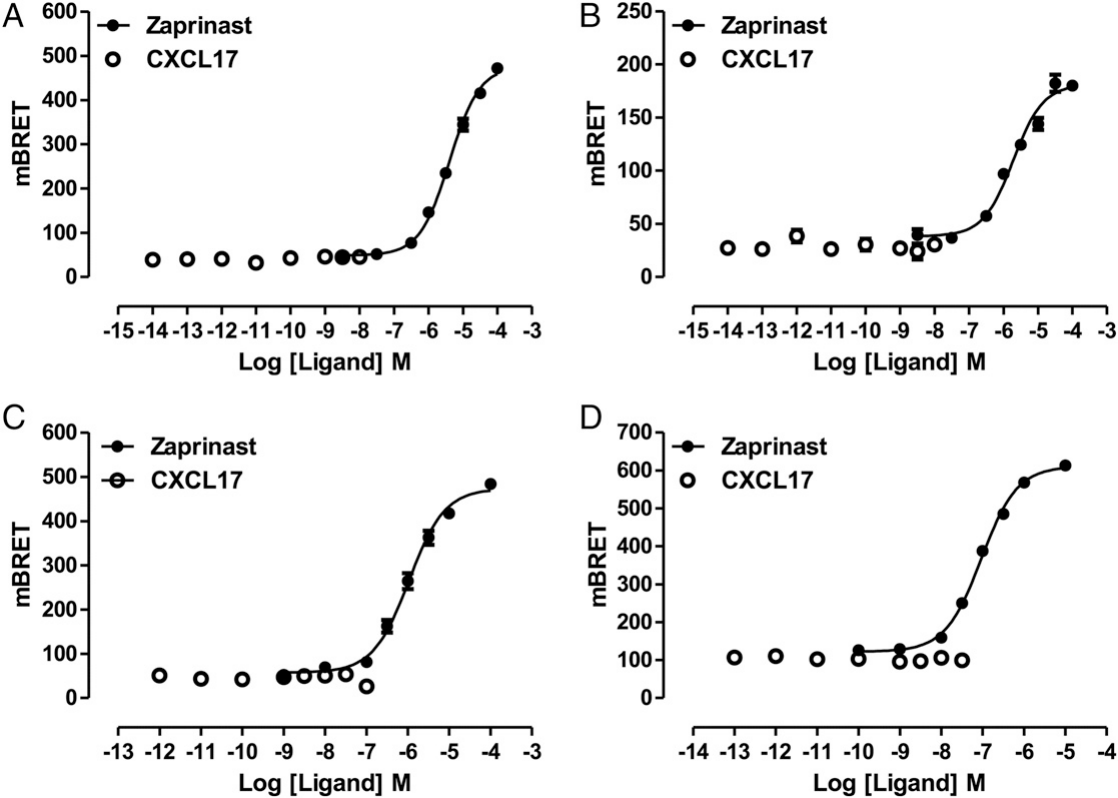
Zaprinast, but not CXCL17, is able to promote interactions between β-arrestin-2 and isoforms and species orthologs of GPR35. Following coexpression of C-terminally eYFP-tagged forms of human GPR35a (**A**), human GPR35B (**B**), mouse (**C**), or rat (**D**) GPR35 and *Renilla* luciferase-tagged β-arrestin-2, the indicated concentrations of the GPR35 agonist zaprinast (filled symbols) or CXCL17 (open symbols) were added, and induced interactions between GPCR35 and β-arrestin-2 were assessed 5 min later by measuring bioluminescence resonance energy transfer.

The most widely used approach to demonstrate agonist ligand function at GPR35 has been to measure induced recruitment to the receptor of a β-arrestin isoform (30–33). Following cotransfection into HEK293 T cells of the short isoform of human GPR35 (GPR35a), tagged at the C terminus with enhanced yellow fluorescent protein (eYFP), along with a C-terminally *Renilla* luciferase-tagged form of β-arrestin-2, the well-characterized synthetic GPR35 agonist zaprinast (22, 30, 32, 34) promoted interactions between the receptor and the arrestin in a concentration-dependent fashion (Fig. 1A), with the negative logarithm of the EC_50_ (pEC_50_) = 5.46 ± 0.04 (mean ± SEM, *n* = 3). In contrast, at concentrations up to 100 nM, CXCL17 was entirely without effect (Fig. 1A). Human GPR35 exists as two isoforms, with the longer version possessing an extended N-terminal domain comprising an additional 31 aa (16). As binding of chemokines to their cognate receptors often involves key interactions within the receptor N-terminal domain (35, 36), we next tested whether this longer isoform of human GPR35 (GPR35b) would recruit β-arrestin-2 in a CXCL17-dependent manner. The chemokine was also entirely inactive in these experiments (Fig. 1B), whereas, once again, zaprinast was an effective agonist (Fig. 1B), with pEC_50_ = 5.77 ± 0.10 (mean ± SEM, *n* = 3). To assess whether CXCL17 might have effects at rodent orthologs of GPR35, experiments akin to those described above were performed using either mouse (Fig. 1C) or rat GPR35 (Fig. 1D). In these species, only a single isoform has been identified, and this corresponds to human GPR35a (18). However, once more, CXCL17 was inactive, whereas at each rodent ortholog zaprinast was an effective agonist. As reported previously (32), zaprinast was more than 10-fold more potent at rat GPR35 (pEC_50_ = 7.04 ± 0.03) than the mouse (pEC_50_ = 5.98 ± 0.05) form.

**FIGURE 2.**
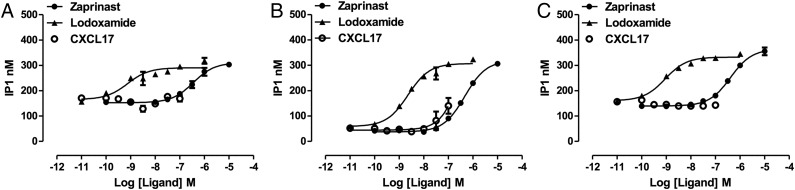
CXCL17 is unable to promote G protein–mediated signaling via isoforms of human GPR35. Either the short, GPR35a (**A** and **B**), or the N-terminally extended, GPR35b (**C**), isoforms of human GPR35 were expressed in HEK293 cells along with the chimeric G protein α subunits G_q_–G_13_ (A) or G_q_–G_i1/2_ (B and C). Following incubation with the indicated concentrations of zaprinast (filled circles), lodoxamide (filled triangles), or CXCL17 (open symbols) for 30 min, levels of inositol phosphates were measured.

Interaction with a β-arrestin reflects initial steps in desensitization and internalization of a GPCR rather than the canonical route(s) of G protein–mediated signal transduction. To assess whether CXCL17, although unable to promote interactions with a β-arrestin, might still activate GPR35 but act as an entirely G protein–biased (37) agonist, we coexpressed human GPR35a alongside chimeric G protein α subunits (38, 39) consisting of the backbone sequence of the G protein G_q_, which allows downstream activation of phospholipase β1 and the hydrolysis of the membrane phospholipid phosphatidylinositol 4,5 bisphosphate but in which the C-terminal 5 aa are replaced by the corresponding residues from either G_13_ or G_i1/2_, as these are the receptor-interacting residues of the G proteins that GPR35 has previously been shown to be able to couple to (20). When employing the G_q_–G_13_ chimera, measures of inositol phosphate production showed that both zaprinast (pEC_50_ = 6.57 ± 0.05) (Fig. 2A) and the high-potency agonist lodoxamide (22) (pEC_50_ = 9.13 ± 0.24) (Fig. 2A) were able to increase levels of inositol phosphates in a concentration-dependent manner. Once more, CXCL17 was unable to mimic these effects (Fig. 2A). Equivalent results were obtained when using the G_q_–G_i1/2_ chimera (Fig. 2B) except at the very highest concentration of CXCL17 used (100 nM), in which a very minor increase in inositol phosphate production compared with the increase produced by either zaprinast (pEC_50_ = 6.33 ± 0.03) or lodoxamide (pEC_50_ = 8.64 ± 0.08) was detected (Fig. 2B). However, when using GPR35b in concert with the G_q_–G_i1/2_ chimera, no response to CXCL17 could be detected (Fig. 2C), whereas both zaprinast (pEC_50_ = 6.39 ± 0.05) and lodoxamide (pEC_50_ = 9.05 ± 0.07) remained efficacious.

Overall, these studies indicate that at least in heterologous transfected cell systems, CXCL17 in unable to act as an agonist for either the long or short isoforms of human GPR35 or to function at the corresponding rat and mouse orthologs.

**FIGURE 3.**
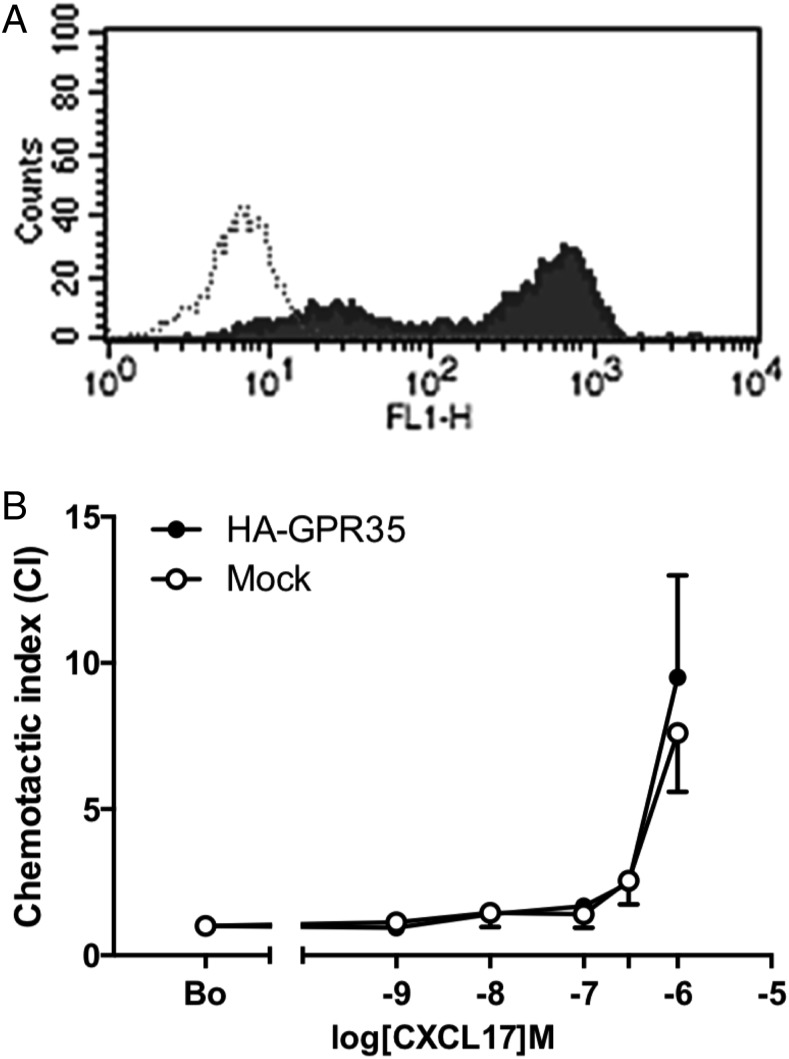
GPR35 is readily expressed in L1.2 cells but does not mediate chemotactic responses to CXCL17. (**A**) represents typical staining profiles obtained for L1.2 cells transiently transfected with the 3xHA GPR35 plasmid. Staining with isotype control is shown as an open histogram, and anti-HA staining is shown as a filled histogram. (**B**) shows the migratory responses of GPR35 expressing L1.2 cells and naive L1.2 cells to increasing CXCL17 concentrations in a modified Boyden chamber. Data are shown as the mean ± SEM from four experiments.

Because the mouse pre–B cell line Ba/F3 had been shown previously to be successfully transfected with GPR35, we switched to express the receptor in the mouse pre–B cell line L1.2., which we have previously used to good effect in expressing chemokine receptors (25). L1.2 cells were transiently transfected with a 3XHA-GPR35 plasmid, and flow cytometric analysis using an anti-HA Ab found the construct to be very well expressed on the cell surface (Fig. 3A). In modified Boyden chamber assays, there was little difference in the chemotactic responses between naive and 3XHA-GPR35 transfectants (Fig. 3B). Both GPR35 transfectants and naive L1.2 cells showed migratory responses to 1 μM CXCL17 that were not significantly different from each other. No chemotaxis at CXCL17 concentrations of 100 nM or below was observed, in keeping with the pharmacological data observed in HEK293 T cells (Figs. 1, 2).

**FIGURE 4.**
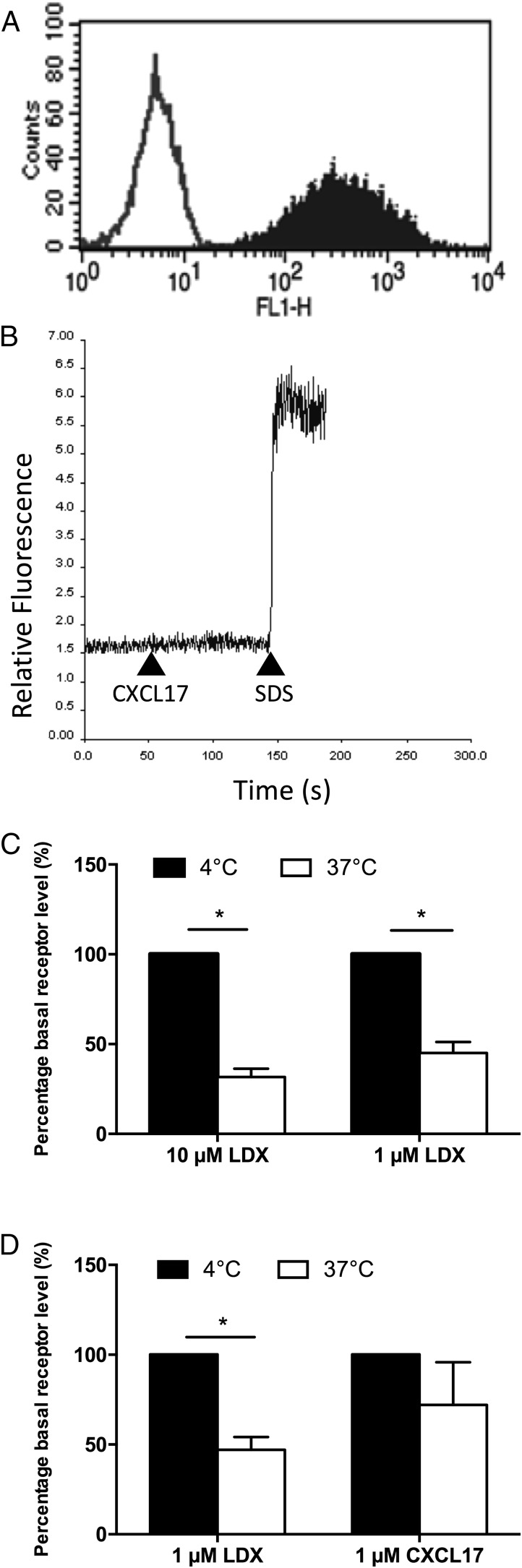
GPR35 is endocytosed by lodoxamide but not by CXCL17. (**A**) shows aAnti-HA staining of clone 23, an L1.2 line stably expressing 3xHA-GPR35 (solid histogram) with isotype control staining shown as an open histogram. (**B**) shows a lack of intracellular calcium flux in response to 100 nM CXCL17 in clone 23 cells. 0.1% SDS was used as a positive control to lyse cells and show successful loading with the Fura-2 dye. The data shown are from a single experiment representative of four experiments. (**C**) shows 3xHA-GPR35 internalization in clone 23 following incubation with different lodoxamide (LDX) concentrations at 4°C or 37°C for 15 min. (*n* = 3). (**D**) shows 3xHA-GPR35 internalization in response to 1 μM lodoxamide or 1 μM CXCL17 incubated at 4°C or 37C for 15 min (*n* = 4). Data are shown as the mean ± SEM for the number of experiments shown in brackets. Statistical differences between controls were confirmed by a Student *t* test. *p < 0.05.

To make our assays as robust as possible, we generated an L1.2 clone, designated clone 23, which stably expressed GPR35 at high levels (Fig. 4A). We then used cells of this clone in assays of chemotaxis, calcium flux, and receptor endocytosis. No significant levels of chemotaxis in response to 1 μM CXCL17 were observed in this line above those observed in the absence of a stimulus (data not shown). Likewise, no calcium flux responses to 100 nM CXCL17 were observed with this line, despite loading of the cells with Fura-2, as denoted by the response to cell lysis with SDS (Fig. 4B). Significant endocytosis of GPR35 in response to 1- and 10-μM concentrations of the high potency GPR35 agonist lodoxamide (22) was observed when cells were incubated at 37°C but not at 4°C, suggestive of clathrin-mediated endocytosis (Fig. 4C). Directly comparing 1-μM concentrations of lodoxamide and CXCL17 in the same assay, we showed that lodoxamide was able to induce significant endocytosis of GPR35, whereas CXCL17 was without activity (Fig. 4D).

**FIGURE 5.**
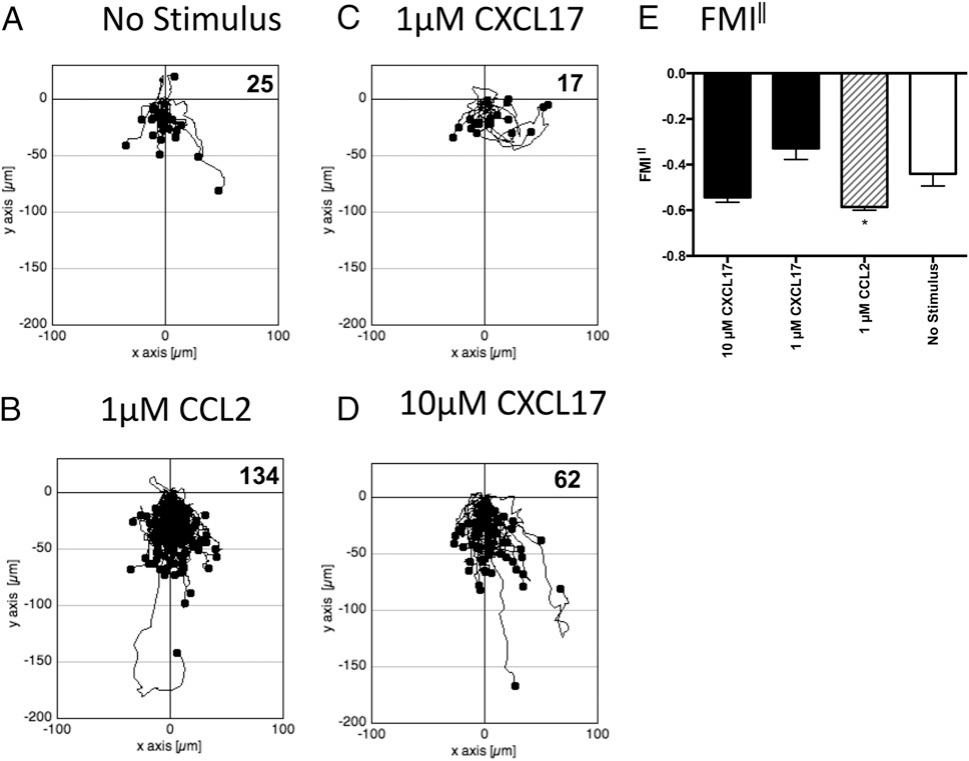
Analysis of the migration of THP-1 cells along chemokine gradients. (**A**)–(**D**) show the paths traveled by individual cells in response to the stimuli indicated. The figures show data pooled from three experiments, with three videos analyzed per condition. Numbers of cells tracked for each condition are shown in the top right-hand corner of the panels. (**E**) shows the mean FMI^‖^ ± SEM of the data shown in (A)–(D). Statistical differences between no stimulus and the indicated stimuli were confirmed by a one-way ANOVA with Bonferroni’s multiple comparisons test. *p < 0.05.

**FIGURE 6.**
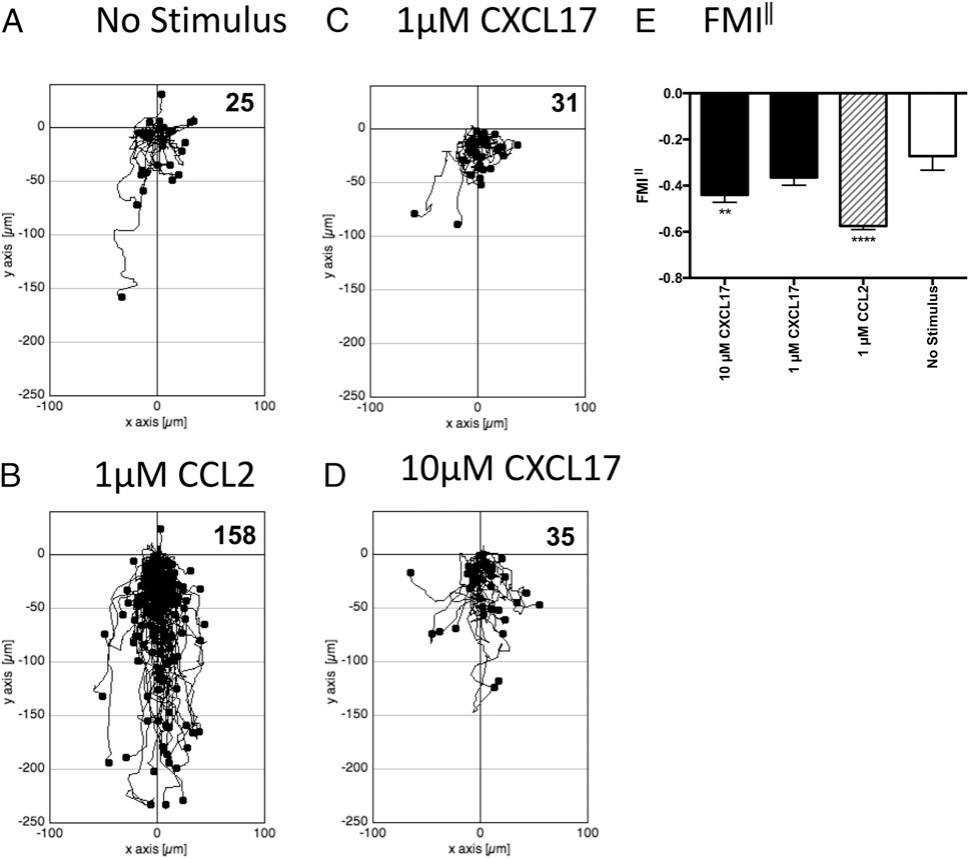
Analysis of the migration of PGE_2_-treated THP-1 cells along chemokine gradients. (**A**)–(**D**) show the paths traveled by individual cells in response to the stimuli indicated. The figures show data pooled from three experiments, with three videos analyzed per condition. Numbers of cells tracked for each condition are shown in the top right hand corner of the panels. (**E**) shows the mean FMI^‖^ ± SEM of the data shown in (A)–(D). Statistical differences between no stimulus and the indicated stimuli were confirmed by a one-way ANOVA with Bonferroni’s multiple comparisons test. **p < 0.01, ****p < 0.0001.

Because it was possible that cells that express GPR35 endogenously also express other proteins lacking in HEK293 T cells that allow CXCL17 to act as a GPR35 agonist, we turned our attention to the study of human THP-1 cells, which were previously shown to be responsive to CXCL17 (23). Initial experiments employed a real-time assay of cell migration (TAXIScan) with resting cells. THP-1 cells were introduced into the chamber and left to migrate for 1 h without exposure to a stimulus or exposed to gradients formed from the introduction of a 1-μl volume of 1 μM CCL2, 1 μM CXCL17, or 10 μM CXCL17. Images were taken at 1 min intervals, and the tracks of the cells from several pooled experiments are shown in Fig. 5. Although little basal migration was observed in the absence of a stimulus (Fig. 5A), many more cells were observed to migrate in response to 1 μM CCL2 (Fig. 5B). Although little cell migration above basal was observed in response to 1 μM CXCL17 (Fig. 5C), approximately three times more cells migrated in response to 10 μM CXCL17 (Fig. 5D). Subsequent analysis of the FMI^‖^ showed a significant response to 1 μM CCL2 and a trend toward significant migration toward 10 μM CXCL17 when compared with the basal levels of migration (Fig. 5E). We subsequently repeated the TAXIScan analysis using THP-1 cells that had been cultured overnight in the presence of PGE_2_ (Fig. 6) because this treatment has been reported previously to enhance chemotactic responses of these cells to CXCL17 (23). In comparison with the basal migration (Fig. 6A), we observed robust migration of cells to 1 μM CCL2 with a 6-fold increase in the number of migrating cells (Fig. 6B). Responses to 1 μM CXCL17 and 10 μM CXCL17 were more modest, although the migration toward 10 μM CXCL17 appeared above that observed under basal conditions (Fig. 6C, 6D). This was confirmed when the FMI^‖^ components of migration were analyzed, with the responses to 1 μM CCL2 and 10 μM CXCL17 significantly greater than the basal response (Fig. 6E). Thus, incubation of THP-1 cells with PGE_2_ increased their capacity to respond by chemotaxis to gradients of CCL2 and of CXCL17.

**FIGURE 7.**
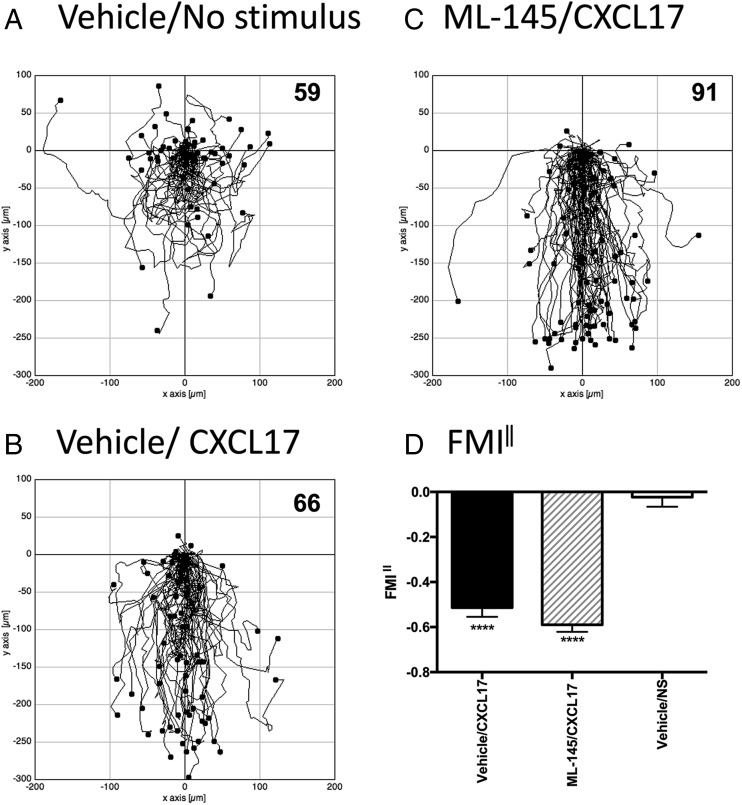
Analysis of the migration of PGE_2_-treated THP-1 cells along CXCL17 gradients following pretreatment with the GPR35 antagonist ML145. (**A**)–(**C**) show the paths traveled by individual cells in response to the stimuli indicated. (A) and (B) show the responses of cells pretreated with the vehicle DMSO, whereas data in (C) show the responses of cells pretreated with the GPR35 antagonist ML145 at a final concentration of 1 μM. Data were pooled from three experiments, with four videos analyzed per condition in each experiment. The total number of cells tracked for each condition are shown in the top right hand corner of the panels. (**D**) shows the mean FMI^‖^ ± SEM of the data shown in (A)–(C). Statistical differences between nonstimulus and the indicated stimuli were confirmed by a one-way ANOVA with Bonferroni’s multiple comparisons test. ****p < 0.0001.

Having established that PGE_2_-treated THP-1 cells could respond to CXCL17 in chemotaxis assays, the question remained whether this was mediated via GPR35, given that Maravillas-Montero et al. (23) had shown that such treatment induces an increase in the levels of GPR35 transcripts. We therefore examined whether the migratory responses of PGE_2_-treated THP-1 cells were sensitive to ML145, a potent, selective antagonist of human GPR35 with an IC_50_ value against EC_80_ concentrations of various GPR35 agonists in the region of 20 nM (32). PGE_2_-treated THP-1 cells were incubated in buffer containing 1 μM ML145 or an identical concentration of the DMSO vehicle, after which they were used directly in TAXIScan assays (Fig. 7). In keeping with our earlier analysis, whereas little directional migration was observed in the absence of a stimulus, significant numbers of cells were observed to migrate in response to 1 μM CXCL17 (Fig. 7A–C). Pre-exposure to the GPR35 antagonist ML145 had no inhibitory effect on the ability of the cells to response to 1 μM CXCL17, which was confirmed by subsequent analysis of FMI^‖^ component (Fig. 7D). Thus, we conclude that although PGE_2_-treated THP-1 cells are able to migrate along gradients of CXCL17, this is unlikely to be mediated by GPR35.

**FIGURE 8.**
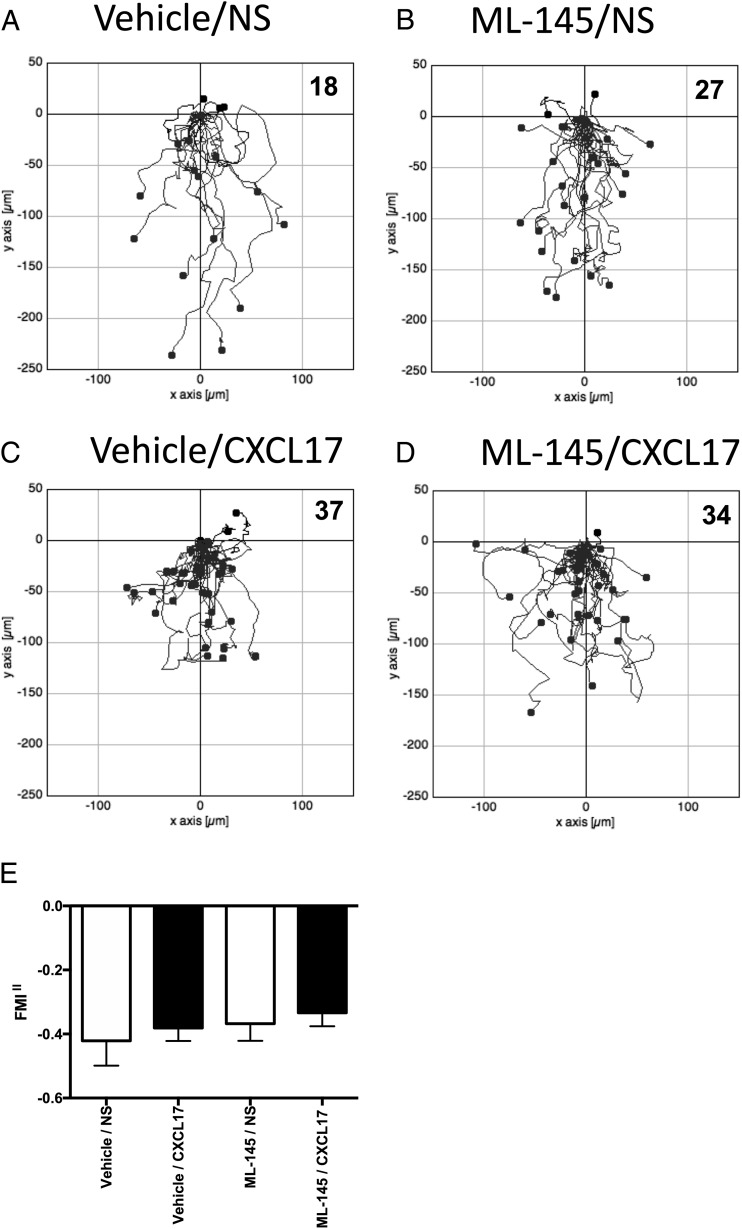
Analysis of the migration of monocytes along CXCL17 gradients following pretreatment with the GPR35 antagonist ML145. (**A**)–(**D**) show the paths traveled by individual cells in response to the stimuli indicated. (A) and (B) show the responses of cells pretreated with the vehicle DMSO, whereas data in (C) and (D) show the responses of cells pretreated with the GPR35 antagonist ML145 at a final concentration of 1 μM. Data were pooled from four experiments, with three movies analyzed per condition in each experiment. The total number of cells tracked for each condition are shown in the top right hand corner of the panels. (**E**) shows the mean FMI^‖^ ± SEM of the data shown in (A)–(D).

Because Pisabarro et al. (8) had previously shown that CXCL17 was chemotactic for CD14^+^ PBMCs, we isolated primary human monocytes and pretreated them with either vehicle (DMSO) or a final concentration of 1 μM ML145 prior to TAXIScan assays using CXCL17 as an attractant (Fig. 8). No significant migration in response to CXCL17 was observed above that seen in the lack of a stimulus (Fig. 8A, 8C), which was unaffected by pretreatment with ML145 (Fig. 8B, 8D, 8E).

**FIGURE 9.**
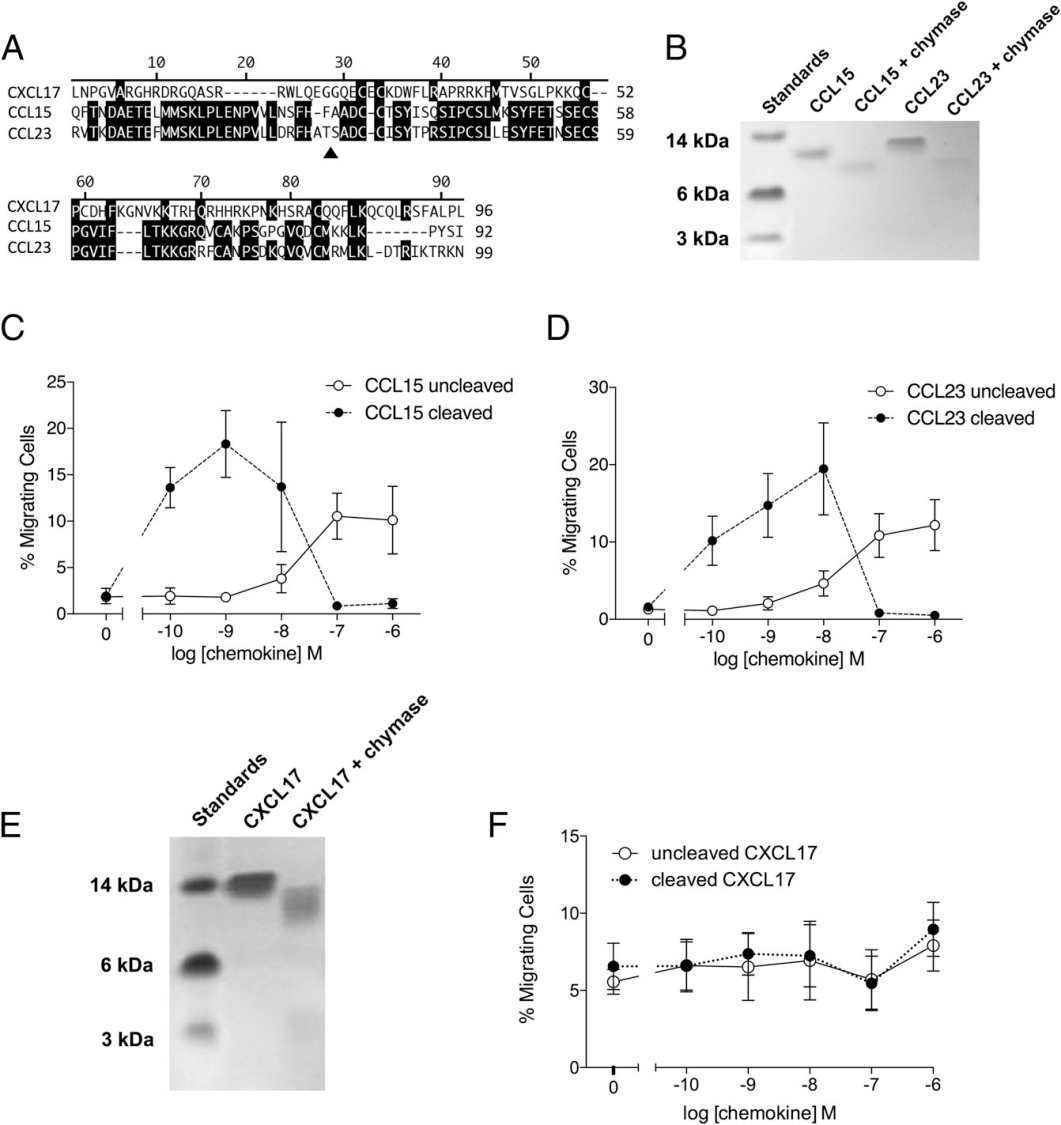
CXCL17 undergoes cleavage by mast cell chymase with little effect upon potency or efficacy in chemotaxis assays. (**A**) shows an alignment of the mature forms of human CXCL17, CCL15, and CCL23. The site of chymase cleavage of CCL15 and CCL23 is shown by a filled triangle. (**B**) shows the cleavage of CCL15 and CCL23 following an 18 hr incubation with chymase. Proteins were separated by SDS-PAGE and visualized by Coomassie Brilliant Blue staining (*n* = 3). (**C**) and (**D**) show the relative potency and efficacy of cleaved and uncleaved CCL15 (C) and CCL23 (D) in Boyden chamber chemotaxis assays using CCR1 transfectants (*n* = 3). (**E**) shows the cleavage of CXCL17 following an 18 h incubation with chymase. Proteins were separated by SDS-PAGE and visualized by Coomassie Blue staining (*n* = 3). (**F**) shows the relative potency and efficacy of cleaved and uncleaved CXCL17 in Boyden chamber chemotaxis assays using PGE_2_-treated THP-1 cells (*n* = 4).

CXCL17 is unusual among chemokines in that the mature protein (devoid of the signal peptide) contains an extended N terminus, as also observed with the CC chemokines CCL15 and CCL23 (Fig. 9A). Because the potency of CCL15 and CCL23 is significantly increased by N-terminal cleavage (40), we assessed whether this was also true for CXCL17. Mast cell chymase was chosen as a potential proteolytic activator of CXCL17 given the mucosal expression pattern of CXCL17 (11). In agreement with a previous report (40), CCL15 and CCL23 were cleaved following incubation with recombinant human chymase (Fig. 9B), which led to increased potency in chemotaxis assays employing CCR1 transfectants (Fig. 9C, 9D). CXCL17 was also a substrate for chymase (Fig. 9E), although cleavage failed to influence the potency or efficacy of the chemokine in chemotaxis assays employing PGE_2_-treated THP-1 cells across a range of CXCL17 concentrations (Fig. 9F).

## Discussion

CXCL17 was the last of the CXC chemokines to be identified by genomic analysis and was originally reported to be chemotactic for dendritic cells and monocytes (8). The gene encoding CXCL17 resides on chromosome 19q13.2, distinct from the cluster on chromosome 4q13.3 where the majority of the CXC chemokines reside (1). In keeping with its discrete chromosomal location, the functions of this chemokine are poorly understood, although its expression appears to be restricted to mucosal tissues (11). Mice deficient in CXCL17 have been reported to have significantly reduced numbers of macrophages in the lung during homeostasis, whereas injection of CXCL17 i.p. results in increased recruitment of macrophages to the peritoneum (12). Thus, in both loss-of-function and gain-of-function rodent studies, CXCL17 appears to recruit macrophages. The role of elevated levels of CXCL17 reported in the lungs of patients with IPF should serve as a driver for research in this area (11). IPF is a progressive, debilitating disease and is an unmet clinical need, with patients having an average life expectancy of only 3 y from diagnosis (41). Because pulmonary macrophages are thought to drive the pathologic condition (42), the identification of the receptor for CXCL17 might provide a potential therapeutic target, and the recent report that GPR35 is a CXCL17 receptor (23) therefore deserves close scrutiny.

GPR35 mRNA has been reported to be expressed in the lungs, stomach, small intestine, colon, and spleens of both humans and mice (18) and also by leukocytes, including basophils, eosinophils, mast cells (21), and iNKT cells (43). As such, GPR35 has attracted attention as a therapeutic target for the treatment of disorders including hypertension, ulcerative colitis, and asthma (15). The role of GPR35 in this latter disease is of particular interest because a number of medications prescribed to stabilize mast cells (including lodoxamide, bufrolin, amlexanox, and pemirolast) have been found by MacKenzie et al. (22) to activate GPR35 with robust efficacy. Using HEK293 T transfectants expressing various species orthologs of GPR35 or either the short and longer isoforms of human GPR35, we were unable to show any activity of CXCL17 at the receptor in assays of GPR35 activation, including β-arrestin-2 recruitment or IP1 production (Figs. 1, 2). While our manuscript was in revision, Park et al. (44) reported that GPR35 expressed in HEK293 T cells was unable to be activated by CXCL17 as deduced by an AP-TGFα shedding assay, supportive of our data.

Using the L1.2 cell line as a background in which to successfully express an HA-tagged variant of GPR35, we observed migration of GPR35 transfectants at a singular concentration of 1 μM CXCL17, which trended toward significance when compared with basal migration. However, the responses of the parental cell line were of a similar potency and efficacy, suggesting that this weak response was not mediated by the GPR35 introduced into the cells. Presumably, this endogenous CXCL17 receptor is able to recognize the human CXCL17 used in this study, which is 71% identical to the mouse ortholog. In contrast to a previous report (23), we were unable to observe intracellular calcium flux in L1.2 transfectants stably expressing 3xHA-GPR35 in response to a 100-nM concentration of CXCL17. The reasons for this are unclear. It is possible that the L1.2 cell line, although like Ba/F3 a mouse B cell line, does not provide a cellular background in which GPR35 can efficiently couple to the intracellular signaling machinery. This would be in contrast to the many other chemokine receptors we and others have successfully expressed in the L1.2 cell line. Similarly, the N-terminal 3xHA tag used by us, but not by Maravillas-Montero and colleagues, has the potential to interfere with chemokine binding and presentation by GPR35, although again, we and others have expressed a variety of epitope-tagged chemokine receptors with little adverse effect on receptor function.

Using the TAXIScan system to assess cell migration in real time, THP-1 cells trended toward significant levels of migration along gradients generated by the addition of 1 μM CXCL17 to the TAXIScan chamber. This was markedly potentiated by overnight culture with PGE_2_, in agreement with the findings of Maravillas-Montero et al. (23). A previous manuscript reported that rat CXCL17 expressed in HEK293 cells undergoes additional N-terminal cleavage, generating an N-terminally truncated species with increased efficacy in chemotaxis assays (45). Given that CXCL17 is associated with mucosal tissues and that mast cell chymase has been shown to cleave the N-termini of the chemokines CCL15 and CCL23 and increase their potency and efficacy (40), we tested the postulate that chymase might enhance the potency and efficacy of CXCL17 for PGE_2_-treated THP-1 cells. However, despite obvious cleavage of CXCL17 by mast cell chymase, we failed to see any significant effect of cleavage upon the potency or efficacy of CXCL17 in Boyden chamber chemotaxis assays. It remains to be seen whether other proteases are able to cleave CXCL17 into a more potent, active form.

Freshly isolated monocytes were unresponsive to CXCL17 in TAXIScan assays, in keeping with the need to treat the THP-1 cells with PGE_2_ to observe robust migration. However, this is in contrast to a previous report in which CD14^+^ cells from a mixture of PBMCs were reported to be recruited by 250-nM–1-μM concentrations of CXCL17 (8). It should be noted that the recombinant CXCL17 used in that study was generated in house and, unlike the CXCL17 used in this study, contained a His tag, which may have influenced receptor binding. Curiously, the authors of that study reported that PGE_2_ treatment of monocytes significantly reduced the responses of cells to their recombinant CXCL17, in stark contrast to the observations of us and Maravillas-Montero and colleagues. McCully et al. (46) have previously shown that PGE_2_ is an essential component of the epidermis-conditioned media, responsible for increasing T cell responses to the chemokine CCL1 and subsequent homing of these cells to the skin. The effects of PGE_2_ in that experimental system are mediated by the EP4 receptor and result in elevated intracellular cAMP levels, which can be mimicked by culture of the cells with forskolin. In those studies, in combination with metabolites of vitamin D_3_, PGE_2_ had the effect of increasing the cell surface expression of CCR8, the cognate receptor for CCL1 (46).

It is tempting to speculate that in our system, the overnight culture of THP-1 cells with PGE_2_ results in elevated levels of a CXCL17 receptor and increased chemotactic responsiveness to CXCL17. Indeed, this was the postulate of Maravillas-Montero and colleagues when they showed that GPR35 mRNA levels were elevated by such treatment. However, in that study, the authors failed to directly show that the induction of GPR35 was directly responsible for the increased responsiveness to CXCL17 (23). In our study, using a well-characterized antagonist of GPR35 that has nanomolar potency at GPR35, we were able to show that chemotaxis of PGE_2_-treated THP-1 cells toward a source of CXCL17 was unimpeded by GPR35 blockade. These data are also in agreement with those of Park et al. (44), who, in a recent study using the alternative small molecule GPR35 antagonist CID2745687, showed that THP-1 cell migration to CXCL17 was independent of GPR35. Taken together with our transfectant data, which was overwhelmingly negative in terms of finding CXCL17 activity at GPR35, we must therefore conclude that GPR35 is not a bona fide receptor for CXCL17 and that this chemokine remains an orphan. As such, efforts to identify the CXCL17 receptor should be resumed with intensity.
